# Variation in Laboratory Reports: Causes other than Laboratory Error

**DOI:** 10.31729/jnma.6022

**Published:** 2022-02-28

**Authors:** Santosh Pradhan, Keyoor Gautam, Vivek Pant

**Affiliations:** 1 Department of Biochemistry, Samyak Diagnostic Pvt. Ltd, Lalitpur, Nepal; 2 Department of Pathology, Samyak Diagnostic Pvt. Ltd, Lalitpur, Nepal

**Keywords:** *diagnosis*, *laboratory*, *report*

## Abstract

When a sample of an individual is measured at different times at the same or different clinical laboratory, the results are always different, even the state of health of an individual is the same. This disparity in the results from clinical laboratories might confuse diagnosing, treating, and monitoring disease. Patients and healthcare professionals usually interpret these differences as laboratory errors. However, this might not always be the case, because laboratory test results are highly variable and are neither consistent nor comparable due to several reasons other than laboratory error, namely pre-analytical variation, biological variation, and analytical variation.

## INTRODUCTION

The ultimate goal of the clinical laboratory is to provide accurate and actionable laboratory results that help healthcare professionals to make appropriate diagnostic or therapeutic decisions, hence improving patient outcomes. However, this has always been a herculean task for clinical laboratories. The challenge gets bigger when the question comes about the release of uniform results. The trend in cross-checking and seeking the second opinion in laboratory results is increasing the dilemma rather than enforcing the results, as most of the time the reports released by two laboratories for the same individual (reproducibility) is different and even the reports released by the same laboratory (repeatability) shows variations.^1^ Shortterm (within-day to two weeks) repeatability and short-term reproducibility are more of a concern than long terms (three to six months). This disparity in the results from clinical laboratories has always been an issue and might cause confusion to diagnose, treat and monitor disease.

## DISCUSSION

Patient and healthcare professionals presume that clinical laboratory results of samples from the same individual at the same day or close timeframe released by the same or different laboratories must be similar or at least comparable otherwise they usually interpret the difference as laboratory error.^2^ Though, this might not always be the case, because laboratory test results are highly variable and are neither consistent nor comparable, as a virtue of a number of reasons other than laboratory error.^3^

Variation in laboratory results is possible at any stage of the total testing process which includes the pre-pre-analytical phase, pre-analytical phase, analytical phase, post-analytical phase, and post-post-analytical phase.^4^ All laboratories are prone to errors and mistakes at the aforementioned phases. Even the laboratory overcomes all these errors and mistakes, variations in results still exist. And these variations in laboratory results can be summed up as pre-analytical variation, biological variation, and analytical variation.^5^

## PRE-ANALYTICAL VARIATION

Even after the standardization of all potential sources of laboratory error, including specimen collection, transportation, preparation, and storage in the pre-analytical phase, considerable variability in the test results does exist. Patient preparation is the commonest pre-analytical factor for this variation. Diet, physical activity, and timing of sampling are major among them that can all have a pre-analytical influence on laboratory results.^6^

Ingestion of certain food (Caffeine, alcohol, nicotine, fruits) before the laboratory test has a huge impact on various analytes. Some food shows a short-lasting effect whereas others have a long-term effect. Variation in laboratory results is likely if the test is performed with and without taking such food, especially those with short-lasting effects, at different instances.^7^

Moderate to strenuous exercise has direct effects on the concentration of analytes like aspartate aminotransferase, lactate dehydrogenase, creatinine kinase, and aldolase due to skeletal muscle release. Intense exercise is also linked with altered thyroid function. There are several other analytes that alter with mild to extreme exercise. It has also been proved that concentrations of various analytes show cyclic variation during the course of the day. Serum iron levels may change from 30% to 50% within a day. Similarly, growth hormone, testosterone levels peak in the morning whereas evening levels are substantially low.^7,8^

Laboratory professionals routinely identify pre-analytical errors and take corrective measures. However, some pre-analytical errors including patient preparation are not under the direct control of the clinical laboratory hence could not be detected at the laboratory end, which might therefore cause a significant impact on test results.^9^

## BIOLOGICAL VARIATION

Every individual has their own homeostatic setting point for every analyte measured. Biological variation is the physiological fluctuation of analyte concentrations around this homeostatic set point. This variation can be of three types, namely; variation over the life (growth, age, menopause), a cyclical variation that can be daily, monthly or seasonal, and random variation. Random variation can be within-subject or intraindividual and between-subject or inter-individual. Within-subject biological variation is defined as the physiological random fluctuation for each individual. And this variation is responsible for the difference in concentration of analytes in each measurement.^10^

## ANALYTICAL VARIATION

Analytical variation occurs due to differences in testing methods and equipment, which results in different analyte values each time they are measured. There can be variations even in the same sample run by the same instrument at close time intervals as all analytical techniques have inherent random variation termed as the analytical imprecision.^11^

Nonetheless, as these variations (within-subject biological variation and analytical variation) are inevitable, how much variation due to these factors is acceptable must be advocated. Reference Change Values (RCV) is one of the objective tools for the assessment of the significance of differences in serial results from an individual.^12^ RCV provides the critical difference between two results that must be exceeded for a significant change to occur and calculated as, RCV= 2^1/2^ × Z × (CV_A_^2^+ CV_I_^2^)^1/2^, where Z is the number of standard deviations appropriate to the probability, (Commonly used Z values are 1.96 and 2.56), CV_A_ is analytical variation and CV_A_ is biological variation.^13,14^

As all laboratories have their analytical imprecision (CV_A_) and database of within-subject biological variation (CV_I_) are available for a large number of analytes (www.westgard.cpm/biodatabase1.htm), calculation of RCV is straightforward.^15,16^ If the difference of two results is less than the calculated RCV for the particular analyte then the variation is not much of clinical significance and should be interpreted as expected variation due to the inherent random sources i.e. biological and analytical variations.^13^

For instance, if the analytical imprecision CV_A_ for serum Glucose is 2.7% and the biological variation CV_I_ (taken from the database) is 5.6% then the RCV for Glucose, 2^1/2^ × 1.96 × (2.7^2^ + 5.6^2^)^1/2^ is 17%. So the changes in Glucose greater than 17% are significant and changes less than this is expected due to the inherent random sources of variation. However, to minimize confounding effects, analytical imprecision CV_A_ should be less than one-half the within-subject biological variation CV_I_^1^ for example CV_A_ (2.7%) is less than 1/2 of CV_I_ i.e. 5.6%.

## WAYS FORWARD

It is rare to get the same results when a sample of the individual is measured at different times, even if the state of health in the individual has not been changed. If such difference in test results from repeated testing is observed, physicians and health care professionals should also be aware of possible factors other than laboratory error. Communication between the physician and laboratory professionals while interpreting such results within the context of pre-analytical, biological, and analytical variations is always imperative.
